# THE ASSOCIATION OF POOR OLFACTION WITH RISK OF CORONARY HEART DISEASE: THE ARIC NEUROCOGNITIVE STUDY

**DOI:** 10.1093/geroni/igae098.1073

**Published:** 2024-12-31

**Authors:** Keran Chamberlin, Chenxi Li, Anna Kucharska-Newton, Zhehui Luo, Mathew Reeves, Srishti Shrestha, David Couper, Honglei Chen

**Affiliations:** Michigan State University, United States; Michigan State University East Lansing, East Lansing, Michigan, United States; University of North Carolina at Chapel Hill, Chapel Hill, North Carolina, United States; Michigan State University East Lansing, East Lansing, Michigan, United States; Michigan State University East Lansing, East Lansing, Michigan, United States; University of Mississippi Medical Center, Jackson, Mississippi, United States; University of North Carolina, Chapel Hill, North Carolina, United States; Michigan State University, Michigan State University, Michigan, United States

## Abstract

Poor olfaction may be associated with cardiovascular health in older adults, but empirical evidence is limited. We evaluated the association of olfactory status with risk of coronary heart disease (CHD) among 5142 participants free of CHD from the ARIC Neurocognitive Study (75.4±5.1 years, 62.9% female, and 23.9% Black participants). Olfaction was assessed in 2011-2013 using the 12-item Sniffin’ Sticks odor identification test, and defined as good (score 11-12), moderate (9-10), and poor (0-8). At-risk participants were followed from baseline to the date of the first outcome of interest, death, last contact, or December 31, 2020, whichever occurred first. We used the discrete-time sub-distribution hazard model to estimate the cumulative incidence of CHD across olfaction groups and adjusted risk ratio (aRR), accounting for covariates, attrition, and the competing risk of death. After up to 9.6 years of follow-up, we identified 280 (5.4%) incident CHD events. Compared with good olfaction, poor olfaction was associated with a higher CHD risk during the first 6 years of follow-up, though the strength of the association gradually attenuated from aRR of 2.05 (95% confidence interval [CI]: 1.02, 4.48) at 2-year follow-up to 1.58 (1.12, 2.34) at 6-year. After 6 years of follow-up, the association continued to diminish until it reached the aRR of 1.07 (0.77, 1.43) at 9.6-year follow-up. Our study suggests that poor olfaction is associated with an elevated CHD risk in older adults, but the association may be lost with extended follow-up.

